# Effects of Fiber Orientation on the Coefficient of Thermal Expansion of Fiber-Filled Polymer Systems in Large Format Polymer Extrusion-Based Additive Manufacturing

**DOI:** 10.3390/ma15082764

**Published:** 2022-04-09

**Authors:** José Luis Colón Quintana, Lucinda Slattery, Jon Pinkham, Joanna Keaton, Roberto A. Lopez-Anido, Keith Sharp

**Affiliations:** 1 Advanced Structures and Composites Center (ASCC), University of Maine, Orono, ME 04469, USA; keith.sharp@maine.edu; 2 Department of Physics and Astronomy, University of Maine, Orono, ME 04469, USA; lucinda.slattery@maine.edu; 3 Department of Civil and Environmental Engineering, University of Maine, Orono, ME 04469, USA; jon.pinkham@maine.edu (J.P.); joanna.keaton@maine.edu (J.K.)

**Keywords:** additive manufacturing, thermo-mechanical characterization, thermoplastic, extrusion, thermal expansion

## Abstract

Large format polymer extrusion-based additive manufacturing has been studied recently due to its capacity for high throughput, customizable bead size and geometry, and ability to manufacture large parts. Samples from three fiber-filled amorphous thermoplastic materials 3D printed using a Masterprint 3X machine from Ingersoll Machine Tools were studied, along with their neat counterparts. Characterization techniques included thermogravimetric analysis (TGA), differential scanning calorimetry (DSC), and thermo-mechanical analysis (TMA). TGA results showed that the fillers decreased the degradation temperature for most of the materials investigated, with a 30 °C decrease for polycarbonate (PC) and a 12 °C decrease for polyethylene terephthalate glycol (PETG). For all the materials used, heat capacity increases with increasing temperature. Moreover, results show that a highly conductive filler increases the heat capacity. In contrast, a material with a lower conductivity decreases the heat capacity indicated in the 15.2% and 2.54% increase for acrylonitrile butadiene styrene (ABS) and PC and a 27.68% decrease for PETG. The TMA data show that the printed bead exhibits directional properties consistent with an orthotropic material. Smaller strains and coefficient of thermal expansion (CTE) were measured along the bead direction and across the bead compared to the through bead thickness showing that fillers are predominantly oriented in the bead direction, which is consistent with the literature. CTE values through bead thickness and neat material are similar in magnitude, which corresponds to the CTE of the matrix material. The experimental results serve to characterize the effect of fiber filler on the part thermal strains in three principal directions and two-part locations during the extrusion and bead deposition of large-format polymer extrusion-based additive manufacturing technologies.

## 1. Introduction

Large-format additive manufacturing technologies have been explored and developed within the last ten years due to their capacity for high throughput and their ability to manufacture objects of a much larger size than 1 m${}^{3}$. These large-scale 3D printers have throughputs above 60 kg/h (132 L/h), compared to desktop additive manufacturing (AM) machines, which typically have less than 1 kg/h (2.2 L/h). This throughput allows for faster printing time for medium-sized objects and makes it possible to manufacture large-scale objects within a reasonable time frame of one to three days. In large-format polymer extrusion-based AM, which is the focus of this study, pellets of the chosen thermoplastic polymer system are first dried to remove any excess moisture that could compromise the integrity of the finished part. The pellets are then fed to the extruder mounted in a gantry system or a robotic arm. In the extruder, the pellets are compacted (solid conveying zone), melted (transition zone), and then pumped (metering zone), creating the pressure to extrude using a corresponding nozzle. The material is then extruded on a layer-by-layer basis to form the desired part. The extrusion head can utilize a screw- or plunger-based mechanism to extrude the material. Moreover, fillers can be added to the matrix to improve the material’s mechanical performance. The benefit of using fillers is that mechanical performance is improved with filler percentage and fiber length.

When exposed to high temperatures, polymers are prone to thermal degradation and softening, affecting the structural properties of a printed part. The mechanical properties of the polymers in question are strongly temperature-dependent. For this reason, it is of great importance to understand and characterize the thermo-mechanical behavior of fiber-filled systems within the manufacturing temperatures. Once characterized, using thermo-mechanical properties in finite element models (FEM) can allow the structural responses of the part to be accurately predicted, saving time and resources, given that each print can take hours to days to finish. It is important to characterize the material to accurately model material behavior and response to attain good numerical simulation results. Work has been done to model the orientation of short fiber polymer composites [1], inter-layer contact and contact pressure [2], and mechanical properties [3]. Moreover, residual stresses, warpage and deformation [4,5], and mesostructure formation [6] have been successfully modeled to understand and predict the manufacturing process.

Fiber-filled polymers are frequently used in large-format AM due to the increase in stiffness they provide and low coefficient of thermal expansion (CTE) arising from the fiber material [7,8]. This is important to ensure dimensional stability and to reduce warping and distortion in the part [9,10], particularly in large-scale printed parts where distortions are often magnified. For this reason, it is essential to perform thermo-mechanical characterization of short fiber-reinforced polymers for use in large-scale additive manufacturing technologies [11] to allow implementation in FEM programs.

**Figure 1:**
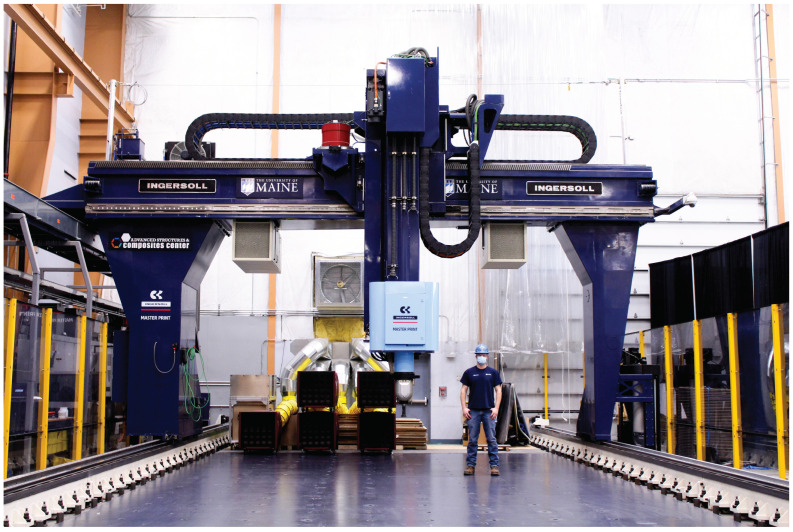
Image comparing the scale of the Masterprint 3X located at the University of Maine with an operator.

**Figure 2:**
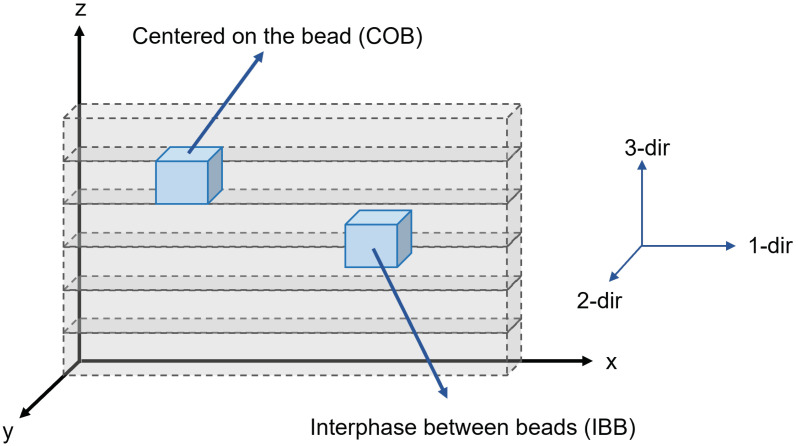
Schematic of TMA sample location. Samples were machined centered on the bead (COB) and at the interphase between beads (IBB). The TMA test was performed along the bead direction (1-dir), across the bead direction (2-dir), and through the bead thickness (3-dir).

For this study, the Masterprint 3X (Ingersoll Machine Tools, Inc., Rockford, IL, USA) large-format 3D printer at the University of Maine in Orono was used to manufacture parts. This 3D printer, the world’s largest [12], has a building envelope of 18.2 m (60 ft) in length, 6.70 m (22 ft) in width, and 3.05 m (10 ft) in height. Figure 1 shows the scale of the equipment by comparing it to an operator. Its extruder can deposit at a rate of 68 kg/h (150 L/h). Three amorphous materials were used for this study. Three characterization techniques, thermogravimetric analysis (TGA), differential scanning calorimetry (DSC), and thermo-mechanical analysis (TMA) were used. The techniques were utilized to determine the degradation temperature, the glass transition temperature, and the heat capacity, and will provide the means to compute the coefficient of thermal expansion (CTE) over a temperature range. The corresponding neat materials were also tested to determine the effect of fiber on the mechanical properties. Moreover, a characterization roadmap is proposed, suggesting the testing procedure for materials used in large format polymer extrusion-based additive manufacturing. This work aims to study and characterize the thermo-mechanical properties of three amorphous fiber-filled thermoplastics commonly used in large format polymer extrusion-based additive manufacturing. In particular, this study focuses on the quantification of the effect of the filler orientation resulting from the manufacturing process by studying the CTE values at different locations and principal directions as schematically depicted in Figure 2.

## 2. Experimental

### 2.1. Materials

This study selected three fiber-filled polymer systems: carbon fiber acrylonitrile butadiene styrene (CF/ABS), carbon fiber polycarbonate (CF/PC), and glass fiber polyethylene terephthalate glycol (GF/PETG). The technical names for each material can be seen in Table 1. The ABS material was selected based on its durability and impact resistance. It also has strong electrical insulation properties, thermal resistance, strength, and machinability [13]. The PC material was selected due to its high strength, high-temperature performance, and higher throughput than ABS and PPE materials. It is also ductile and has a good surface finish. The PETG was selected due to its transparency and its resistance to chemicals and moisture, as well as its good surface finish [14]. This allows the manufacture of functional prototypes such as containers for liquids, signage, and graphic displays. Neat materials were also tested to compare the influence of the filler material on the thermo-mechanical properties.

#### 2.1.1. Manufacturing and Sample Preparation

The Masterprint 3X at the University of Maine was used to manufacture the AM plates. The materials were dried before each printing operation. The CF/ABS was dried at 85 °C for 3–4 h, CF/PC at 125 °C for 4–6 h, and GF/PETG at 54 °C for 3–4 h. The material processing parameters can be found in Table 2. A nozzle diameter of 10.16 mm (0.4 in) was used for the prints with a layer height of 5.08 mm (0.2 in). It is important to mention that the Masterprint 3X used for the prints possesses an unheated bed. This can create residual stresses as a result of the temperature gradient from the unheated bed to the deposited bead and could affect the material molecular and mechanical behavior of the manufactured part [15,16]. Five Novatek Novair 2000 Air Scrubbers (Jon-Don, LLC Headquarters, Roselle, IL, USA) attached to an outside fan were used during the prints. A complete air change of the print volume is performed every 5 min. A floor-to-ceiling curtain encloses the print volume to help maintain a positive pressure zone to help with the air change.

A LAGUNA Swift Computerized Numerical Control (CNC) machine (Laguna Tools, Grand Prairie, TX, USA) was used to machine CTE samples. As the extrusion of a fiber-filled system is a complex phenomenon, specimens were machined at two locations within the printed parts. These are samples centered on the bead (COB) and samples at the interphase between beads (IBB). Testing these locations will give an insight into the CTE response through the printed part. Due to the complex flow exiting the extruder, fiber orientation will be a function of many parameters. Understanding the CTE of the part will allow better predicting capabilities when modeling and manufacturing the part.

A Qixing${}^{\mathrm{TM}}$ Laboratory Mini Hot Press (Wuhan Qien Science & Technology Development CO., Ltd., Wuhan, China) was used to manufacture samples for TMA testing. A total of five layers with an area of 15.24 cm by 15.24 cm were used to manufacture the samples: two stainless steel plates, two Kapton layers, and a mold (Order: plate/Kapton/mold/Kapton/plate). The Kapton material is thermally stable at high temperatures and prevents the material from sticking to the stainless steel plates. Dried pellets were placed on the 10 cm by 10 cm opening of the mold and then placed on the hot press. A pressure of 6.89 MPa (1000 psi) was applied to all three materials. A temperature of 220 °C was used for both ABS and PETG, while a temperature of 270 °C was used for PC. The results will serve as a baseline when compared to the fiber-filled counterparts.

### 2.2. Material Characterization Method

#### 2.2.1. Roadmap

**Figure 3:**
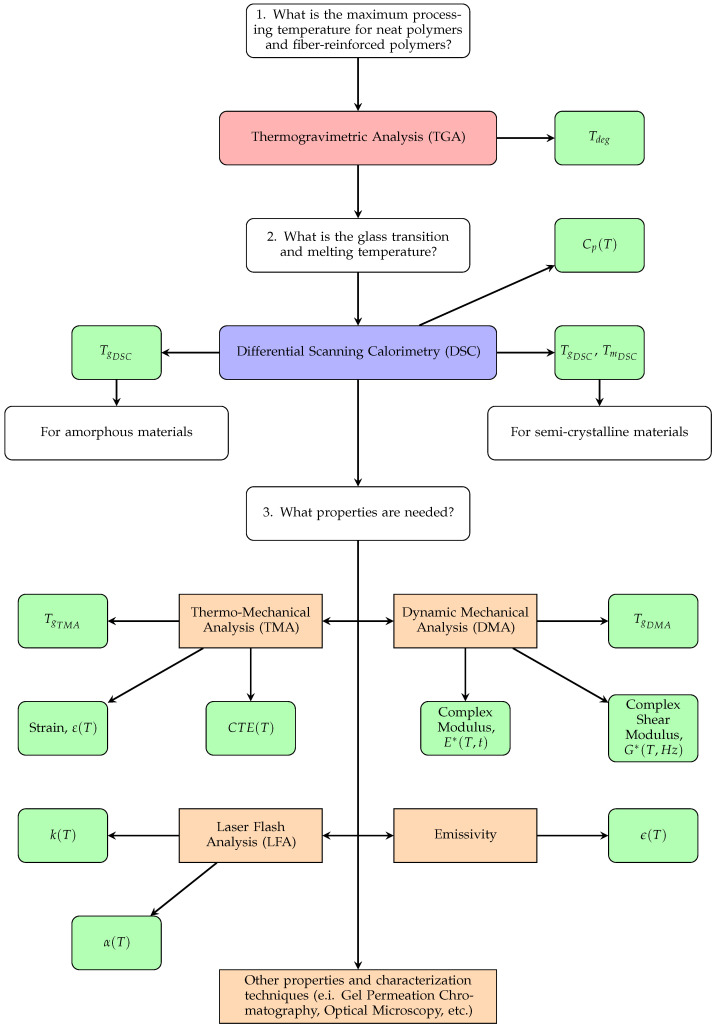
Proposed roadmap for material characterization. Note that a new sample should be used for each characterization method and for each repetition. Labels in color red, blue, and orange represent characterization techniques. Labels in green are material properties acquired from each characterization technique.

Figure 3 shows a proposed roadmap for the characterization of fiber-filled polymer systems in large format polymer extrusion-based additive manufacturing as more educational and research facilities are using this technology [17,18,19]. The characterization methods presented here, although not new, are well understood for a range of material systems. Implementing these materials requires standardization of material characterization to understand material behavior before processing and simulating a part.

As a first step, a thermogravimetric analysis (TGA) is required to measure the mass loss of the material as a function of temperature. This technique provides the degradation temperature and subsequently the upper limit of the processing temperature. Moreover, TGA allows for the determination of the material’s moisture content and filler content, and can analyze the material’s composition. It can also measure volatiles in the polymer material [20].

After performing a TGA, it is necessary to perform differential scanning calorimetry (DSC) on the materials. The DSC measures the heat flow of the material given a temperature input capturing thermal events during heating or cooling. The DSC allows the determination of the glass transition temperature ($T_{g}$), the melting temperature ($T_{m}$), heat capacity, and the percent crystallinity of thermoplastic materials [21]. The $T_{g}$ and $T_{m}$ are important values as some characterization techniques have temperature limitations. For example, some equipment requires the material to be in a solid state, limiting the temperatures at which the material can be tested.

Once the reference temperatures are known ($T_{g}$ or $T_{m}$), different characterization techniques can be utilized. Some of these techniques are laser flash analysis (LFA), emissivity testing, thermo-mechanical analysis (TMA), and dynamic mechanical analysis (DMA). The LFA is used to measure thermal diffusivity. Given the heat capacity, the thermal conductivity can be calculated as a function of temperature [22,23].

The TMA measures material deformation during a heating or cooling event. During the temperature change, the material can contract or expand. Strain versus temperature curves can be obtained from this method. The curves allow the calculation of the coefficient of thermal expansion (CTE) [24]. This value is essential for this application as it can help predict material deformation, residual stresses, and warpage of a printed part. Moreover, due to the fiber alignment during extrusion and deposition, the materials tested in this study exhibited orthotropic properties [25].

The DMA is utilized to measure the dynamic properties of the material. Assuming that the material is thermo-rheologically simple, a time-temperature-superposition (TTS) principle can be used to create a master curve [26,27] to compute its mechanical properties over a range of time and temperature. The technique allows the measurement of creep modulus, relaxation modulus, and elastic modulus, to mention a few.

An additional measure of interest is the emissivity of the filled or unfilled polymer. As emissivity is the measure of an object’s ability to absorb, transmit, and emit infrared energy [28]; this is important when using infrared (IR) cameras. This measurement is important as it helps compare the modeling temperature field with the in-situ temperature results [29].

Utilization of the proposed roadmap will ensure the optimal application of material for any particular process. With this information, a set of techniques can be utilized to characterize the material and understand its mechanical and thermal properties. The work presented here will show the use of TGA and DSC to determine the limit temperature for the TMA test. Then, the effect of fiber-filled polymers will be determined and the CTE values calculated.

#### 2.2.2. Thermogravimetric Analysis (TGA)

A TA Thermogravimetric Analyzer TGA Q500 (TA Instruments, New Castle, DE, USA) was used for the thermal characterization of the materials. For each sample, samples were cut from the AM parts and placed onto a tared platinum pan. The tests were conducted using a heating rate of 10 °C, and a final temperature of 600–700 °C (depending on the material) in a nitrogen gas atmosphere at a flow rate of 40 mL/min following the ASTM E1131-20 standard for thermoplastic materials [30]. A total of three tests were performed for each material to confirm the repeatability of the results. The results were then analyzed using the TA Universal Analysis software.

#### 2.2.3. Differential Scanning Calorimetry (DSC)

A TA Differential Scanning Calorimeter DSC2500 (TA Instruments, New Castle, DE, USA) was used for the thermal characterization of the materials. The DSC procedure consisted of a three-step process, heat-cool-heat. The first heating provides the thermal properties resulting from the manufacturing process. The cooling serves as an annealing process that homogenizes the material to reach equilibrium. The second heating shows the actual properties of the material. TA’s Tzero pans and lids were used for the material characterization. The material (between 3 mg to 10 mg) and pan and lid were weighed. Then, both masses (sample and pan and lid) were input into the program. To have a homogeneous temperature throughout the sample and to follow the ASTM D3418-21 procedure, a ramp of 10 K/min was used [31]. The maximum temperature was selected based on the TGA results. A total of three tests were performed for each material to confirm the repeatability of the results. The results were then analyzed using the TA Universal Analysis software.

#### 2.2.4. Thermomechanical Analysis (TMA)

A TA Thermomechanical Analyzer TMA Q400 (TA Instruments, New Castle, DE, USA) was used for the thermo-mechanical characterization of the materials. The TMA procedure consisted of placing the sample in the specimen holder under the probe, placing the temperature sensor near the sample, applying the initial load force on the sample, and closing the furnace. The test was performed from ambient temperature to 25 °C above the glass transition temperature ($T_{g}$ + 25 °C ) at a ramp of 5 °C/min in accordance with the ASTM Standard E831-19 [32]. A total of three tests were performed for each material and each of the principal directions to confirm the repeatability of results. Samples were tested from two locations within the manufactured part. These were samples centered on the bead and between two adjacent beads. Figure 2 shows a representation of the sample locations. The results were then analyzed using the TA Universal Analysis software.

### 2.3. Volumetric Strains as a Function of Temperature

The volumetric strain is defined as the change in volume divided by the original volume as shown in Equation (1):

(1) $$\varepsilon_{vol}=\frac{\Delta V}{V_{o}}$$

**Figure 4:**
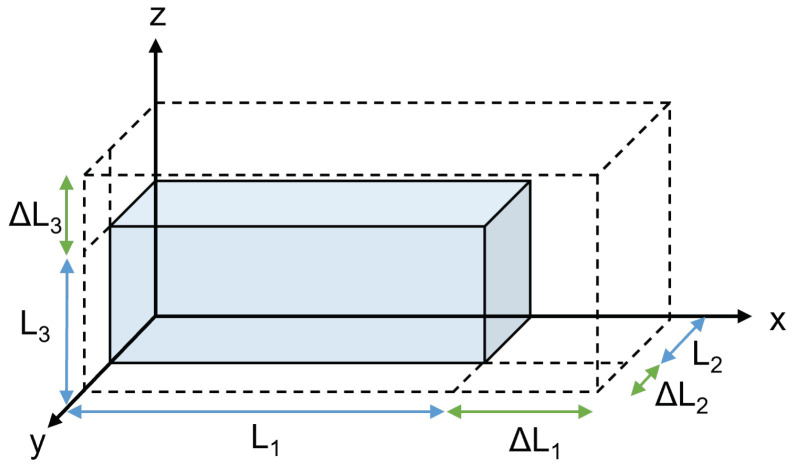
Schematic of material deformation. The variables $L_{1}$, $L_{2}$, and $L_{3}$ correspond to the initial length in the *x*-, *y*-, and *z*-direction, respectively. $\Delta L_{1}$, $\Delta L_{2}$, and $\Delta L_{3}$ correspond to the change in length in the *x*-, *y*-, and *z*-direction, respectively, resulting from the material deformation due to a temperature change.

Due to the orthotropic nature of the short fiber-reinforced composites, the material will deform at different rates with respect to the three principal directions. After deformation, the final volume can be expressed as shown in Equation (2) and is shown in Figure 4:

(2) $$V_{final}=V_{o}+\Delta V=\left(L_{1}+\Delta L_{1}\right)\left(L_{2}+\Delta L_{2}\right)\left(L_{3}+\Delta L_{3}\right)$$

where the initial volume $V_{o}=L_{1}L_{2}L_{3}$, $L_{i}$ corresponds to the length in the corresponding principal direction, and $\Delta L_{i}$ is the change in length of the given principal direction. Rearranging the terms provides an expression for the volumetric strain as shown in Equation (3) [33,34]:

(3) $$\frac{\Delta V}{V_{o}}=\frac{\Delta L_{1}}{L_{1}}+\frac{\Delta L_{2}}{L_{2}}+\frac{\Delta L_{3}}{L_{3}}$$

(4) $$\varepsilon_{vol}=\varepsilon_{1}+\varepsilon_{2}+\varepsilon_{3}$$

Equation (4) shows that the volumetric strain can be approximated by the summation of the strain of each of the principal directions.

## 3. Results and Discussion

### 3.1. TGA Results

**Figure 5:**
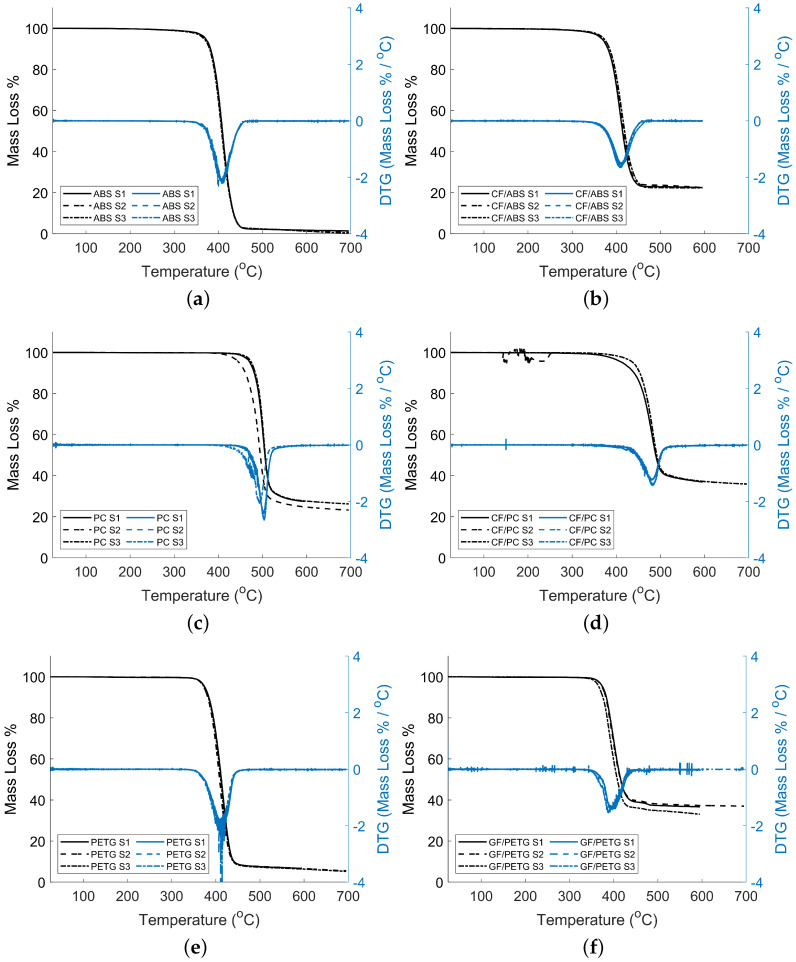
Mass loss % (left axis) and DTG (right axis) of (**a**) ABS; (**b**) CF/ABS; (**c**) PC; (**d**) CF/PC; (**e**) PETG; and (**f**) GF/PETG. S1, S2, and S3 correspond to sample 1, sample 2, and sample 3, respectively.

TGA results using a nitrogen gas atmosphere are presented in Figure 5. The results show the percent mass loss as a function of temperature from ambient temperature (approximately 20 °C) to 600–700 °C. All of the samples displayed a single staged degradation occurring at the onset degradation temperature ($T_{deg-onset}$) and ranging from 367.6 °C to 480.3 °C identified in Figure 5 for singular tests. The average $T_{deg-onset}$ and the peak of the derivative of the mass loss percentage versus temperature curve ($T_{deg-peakDTG}$) of three tests are presented in Table 3 for each material used. The residue of each material is also reported in Table 3.

**Figure 6:**
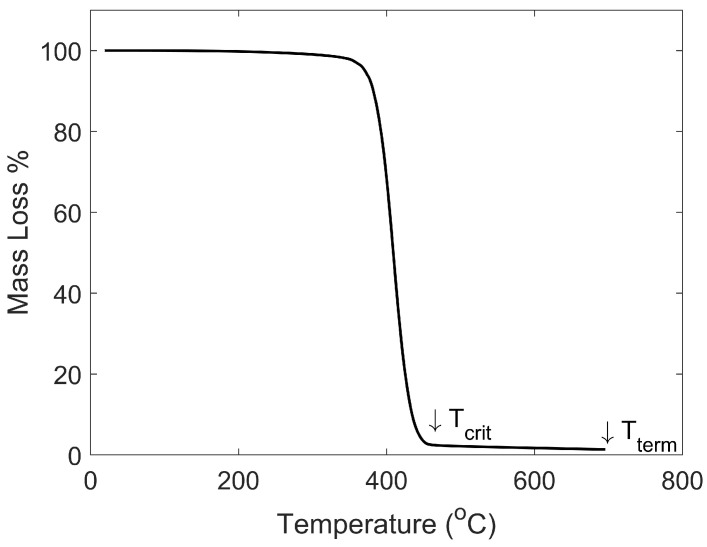
Example Analysis for $T_{crit}$ and $T_{term}$ (ABS). $T_{crit}$ refers to the temperature at the end of the single-stage degradation while $T_{term}$ is the temperature at the end of the test. The mass percentage is reported at both $T_{crit}$ and $T_{term}$ for all the materials used as shown in Table 3.

The critical temperature ($T_{crit}$) was determined in the TA Universal Analyzer as the mass percentage of the sample at the end of the single-stage degradation. The terminal temperature ($T_{term}$) was determined as the mass percentage at the end of the test. For each material, values were averaged for the three runs. An example of analysis for $T_{crit}$ and $T_{term}$ are shown in Figure 6. Complete results are included in Appendix B.

Both the ABS and CF/ABS had single-stage degradation and a remaining mass percentage at the end of the test shown in Figure 5a,b and Table 3. The derivative of the mass loss percentage and temperature curve (DTG) provides insight into the degradation stage. For our material, a singular minimum represents a single-stage degradation. The neat ABS had a $T_{deg-onset}$ of 384.26 ± 1.43 °C with 0.79% remaining at the end of the test. This value is consistent with previous TGA analysis for ABS with $T_{deg-onset}$ between 375 °C and 390 °C as reported by Fong et al. [35]. As reported in Table 3, $T_{deg-peakDTG}$ is 407.79 ± 1.34 °C, showing that, for the neat ABS, the largest change in mass or fastest degradation occurs at this temperature. This peak corresponds to a mass loss of 40%. With the addition of carbon fibers, a decrease in $T_{deg-onset}$ and an increase in percent mass remained at the end of the test. For CF/ABS, a $T_{deg-onset}$ of 384.15 ± 1.69 °C was observed, with a remaining percent mass of 22.46%. The $T_{deg-onset}$ is consistent with results in published literature for CF/ABS of the same fiber percentage, with a $T_{deg-onset}$ between 350 °C and 400 °C, as reported by Billah et al. [36]. The increase in mass percentage remaining at the end of the test can likely be attributed to the CF present having a higher $T_{deg-onset}$ than ABS. The increase in mass percentage from neat to carbon fiber ABS is approximately the fiber percentage added (20%). For CF/ABS, the recorded $T_{deg-peakDTG}$ is 410.77 ± 1.84 °C. This peak corresponds to a mass loss of approximately 40%. With the addition of carbon fiber, there is a negligible difference in $T_{deg-peakDTG}$ given the overlapping uncertainties reported.

Both the PC and CF/PC had single-stage degradation and a remaining mass percentage at the end of the test as shown in Figure 5c,d and Table 3. The DTG plot shows a singular minimum representing a single-stage degradation. Notably, there is slight variation in the minimums of the DTG plot. This can likely be attributed to batch variability and sample uniqueness. A disturbance in Figure 5d is observed for one of the samples, and it is attributed to vibrations of the equipment during measurement. The PC had a $T_{deg-onset}$ of 480.29 ± 9.09 °C, with 25.64% remaining after the test had concluded. This value is consistent with previous TGA analysis for PC with $T_{deg-onset}$ between 400 °C and 500 °C, as reported by Uyar et al. [37]. As reported in Table 3, $T_{deg-peakDTG}$ is 499.29 ± 5.53 °C. This peak corresponds to a mass loss of approximately 40%. The large uncertainty is caused by sample variability, visualized in Figure 5c. With the addition of carbon fibers, a decrease in $T_{deg-onset}$ was witnessed, and an increase in percent mass remaining at the end of the test as shown in Figure 5c,d. For CF/PC, a $T_{deg-onset}$ of 450.91 ± 3.86 °C was observed with a remaining percent mass of 36.25%, which is approximately 10% greater than the neat PC. The $T_{deg-onset}$ is consistent with results in published literature for CF/PC, with a $T_{deg-onset}$ between 425 °C and 525 °C as reported by Hacioglu et al. [38]. Hacioglu et al. [38] produced TGA results for a CF/PC of fiber percentage of 10%, and reported the maximum processing temperature at 514.7 °C. As reported in Table 3, $T_{deg-peakDTG}$ for CF / PC is 481.97 ± 1.07 °C. This peak corresponds to a mass loss of approximately 40%. In comparison with neat ABS accounting for uncertainty, this $T_{deg-peakDTG}$ is approximately 10 °C lower than the neat material.

PETG and GF/PETG had single-stage degradation and a remaining mass percentage at the end of the test as shown in Figure 5e,f and Table 3. The single state degradation is also represented for both neat and fiber-filled PETG by the singular minimum of the DTG plot. The neat PETG had a $T_{deg-onset}$ of 387.61 ± 1.68 °C with 5.85% remaining after the test had concluded. This value is consistent with previous TGA analysis for PETG with $T_{deg}$ between 375 °C and 425 °C as reported by Techawinyutham et al. [39]. As reported in Table 3, $T_{deg-peakDTG}$ is 413.67 ± 3.54 °C. Notably, there is a sharp minimum in the DTG graph. This is caused by the steepness of the percent mass graph, showing a fast degradation once reaching the onset temperature. This peak corresponds to a mass loss of approximately 40%. With the addition of glass fiber, a decrease in $T_{deg-onset}$ and an increase in percent mass remained at the end of the test. For GF/PETG, a degradation temperature of 375.10 ± 3.19 °C was observed, with a remaining percent mass of 35.57%. This is consistent with current literature with a degradation temperature being reported for this material in the range of 350 °C to 400 °C by Lorenzana et al. [40]. The remaining mass percentage at the end of the test is larger than guaranteed by the manufacturer, with a minimum mass percentage expected to be 30%. The increase in mass percentage remaining at the end of the test can likely be attributed to the GF present having a higher $T_{deg-onset}$ than neat PETG. The increase is 29.72%, approximately the material’s glass fiber percentage (30%). For GF/PETG, $T_{deg-peakDTG}$ is 396.24 ± 6.39 °C. This peak corresponds to a mass loss of approximately 40%. In comparison with neat PETG accounting for uncertainty, $T_{deg-peakDTG}$ is approximately 7.5 °C lower than the neat material.

In general, $T_{deg-peakDTG}$ is greater than $T_{deg-onset}$ for all the materials tested. Moreover, on average, there is a 40% in mass loss if $T_{deg-peakDTG}$ is selected as the degradation temperature. This can result in a decrease in properties if a process temperature is selected near $T_{deg-peakDTG}$. A more conservative approach is selecting $T_{deg-onset}$ as the degradation temperature as there is less than 10% in mass loss if a temperature near $T_{deg-onset}$ is used.

### 3.2. DSC Results

#### 3.2.1. Heat Flow

**Figure 7:**
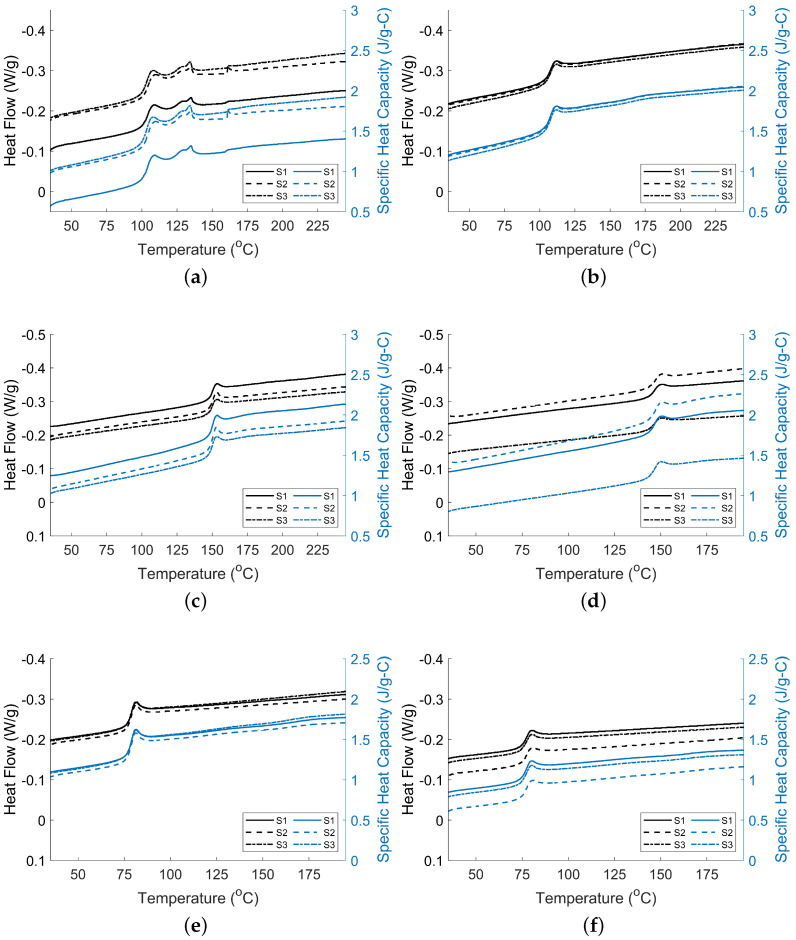
Heat flow (left *y*-axis) and heat capacity (right *y*-axis) of (**a**) ABS; (**b**) CF/ABS; (**c**) PC; (**d**) CF/PC; (**e**) PETG; and (**f**) GF/PETG. S1, S2, and S3 correspond to sample 1, sample 2, and sample 3, respectively.

Figure 7 shows the DSC results for all the materials tested. Both the heat flow and specific heat capacity as a function of temperature are reported. The results were taken from the second heating to represent the material properties after annealing.

In Table 4, the average mid-point $T_{g}$ values are reported for all neat and fiber-filled materials. The mid-point values were determined using TA Universal Analysis software. Romanova et al. [41] reported $T_{g}$ of 152 °C for PC. Tsukuda et al. [42] also stated that ABS has $T_{g}$ of 102 °C. These results are in agreement with the results shown in Table 4, where PC has a $T_{g}$ of 149.00 °C and ABS $T_{g}$ of 104.32 °C. Shi et al. [43] reported a $T_{g}$ value of 85 °C for PETG while Latko-Duralek et al. [44] reported a value of 75 °C for PETG. From this research, a value of 77.51 °C was reported for PETG, which is within the previously reported values. CF/ABS is observed to have an average $T_{g}$ of 106.57 °C similar to 106 °C reported by Billah et al. [11]. CF/PC is observed to have an average $T_{g}$ of 145.35 °C, similar to 154 °C reported by Phua et al. [45]. Lorenzana [40] reports 77.5 °C for GF/PETG compared to the observed value of 75.99 °C in Table 4.

ABS shows signs of two peaks within the DSC results present in Figure 7a. The first endothermic region represents the softening of the partially gelled crystallites. The second endotherm represents the softening of crystallites that did not gel during the heating of the sample, which results in the softening of the material. This behavior is also observed for other amorphous materials [46]. ABS, being an amorphous material, has no set melting point. Previous research shows the role played by unknown materials being present within the ABS matrix and a second peak visible in DSC results [11,36,47]. Variation of the ABS co-polymers can also play a role in the amplitude of the peaks. Reed et al. [47] verified that there are materials left within the ABS from the molding of the SAN and butadiene polymers, which form ABS. The reasoning that CF/ABS does not show a second peak may be due to the difference in material suppliers. The supplier of the CF/ABS may not use the unknown additive to mold the ABS material as previously discussed.

The results show multiple behaviors for $T_{g}$ values when adding fillers to polymer matrices. The behavior is complex and can be caused by many factors. For the materials tested, PC and PETG showed decreases in $T_{g}$ with the addition of fibers, whereas ABS showed an increase. It is worth mentioning that the neat ABS and PC are from different suppliers compared to the fiber-filled material. The effects, although not drastic, are noticeable as PC sees a 2.45% decrease from neat to fiber-filled and PETG sees a 1.96% decrease as seen in Table 4. ABS sees a 2.16% increase from neat to fiber-filled for its reported $T_{g}$ values. The expectation would be that adding fibers would increase material stiffness, viscosity, and strength. Those increases would lead to increases in $T_{g}$. However, the results do not back that expectation for all materials tested. The decrease for PC and PETG can best be described by the effects of the fiber, lowering the $T_{g}$ values. This process is described by Tajvidi et al. [48], which may have tested a semi-crystalline material, but the concept might apply to amorphous materials. Other amorphous material results such as Billah et al. [11] see similar behavior, explaining that additive materials are left behind within the fiber-filled ABS. Moreover, the influence of additives on the matrix material can influence the phase change behavior of polymers, as seen in our results and others [11]. Other research has found $T_{g}$ decreases with the additions of fiber as well and concluded polymer degradation could also affect the $T_{g}$ [49].

For ABS, the increase in $T_{g}$ with the addition of fibers would support the expectation of the fiber addition increasing stiffness and viscosity; therefore, delaying the temperature for the transition from glassy to rubbery phase. Billah et al. [11] reported an increase as well with $T_{g}$ for ABS at 104 °C, while CF/ABS showed the transition at 106 °C. The increase was explained by the carbon fiber reducing the mobility of the polymer chains [11]. However, Billah et al. [36] reported a 5 °C decrease in the $T_{g}$ for ABS when carbon fiber was added, going from 110 °C to 105 °C from neat to fiber-filled. The attributing factor used to explain the phenomena seen by Billah et al. was that fibers enhanced the polymer chains’ mobility [36]. This explains the $T_{g}$ increase observed from the results presented in this test; the fibers hindered the polymer chains’ mobility instead of enhancing it. In both Billah et al. [11,36] works, there was a common report of CF/ABS having $T_{g}$ occur near 105–106 °C, but there was a difference in the ABS showing $T_{g}$ at 110 °C [36] and 104 °C [11]. This shows that the combination of ABS and carbon fiber has consistent thermal properties regardless of the different ABS materials used and their associated properties. The same behavior is shown in Table 4 where CF/ABS has $T_{g}$ at 106.57 °C in agreement with previous reports [11,36] regardless of the neat ABS material.

#### 3.2.2. Specific Heat Capacity

In Table 4, the average specific heat capacity ($C_{p}$) values are shown for a temperature of 35 °C and the maximum testing temperatures (see Table 5). Specific heat capacity is shown to increase as temperature increases in this study as well as in other polymer system studies [50,51,52]. The highest increase was 101.2% for ABS, and the lowest increase was 65.4% for PETG.

In addition, increased specific heat capacities of carbon fiber-filled polymers from their neat state are observed, as seen in the 15.2% and 2.54% respective increases for ABS and PC materials at their maximum temperatures. However, the glass fiber is observed to decrease specific heat capacity based on the 27.68% decrease for PETG from neat to fiber-filled. As described by Tsukuda et al. [42], the addition of glass fiber beads to ABS caused a decrease in specific heat capacity. The decrease in specific heat capacity is linked to the minimal interaction between glass beads and the ABS material resulting from the minimal surface area that beads provide for adhesion. Billah et al. [11] reports $C_{p}$ values of 0.52 J/g-°C for ABS and 2.00 J/g-°C for CF/ABS, both reported at 80 °C, which agree with the results in Table 4. The $C_{p}$ values in Table 4 are similar to those found by others [42,53,54,55]. The increase seen in both can be described by the conductivity of the carbon fiber leading to energy dissipation and increasing the specific heat capacity of the material [11]. Since the specific heat capacity is the amount of energy required for the internal material temperature to increase, if energy is lost through dissipation, it is logical for more energy to be required to increase the internal temperature.

Typically, prediction of the specific heat capacity of a fiber-filled molded polymer can be achieved by using the rule of mixtures in Equation (5) [52]:

(5) $$C_{m}=\psi *C_{f}+\left(1-\psi \right)*C$$

In Equation (5), $C_{m}$ is the composite specific heat capacity, $C_{f}$ is the fiber specific heat capacity, ψ is the weight ratio of fiber, and *C* is the specific heat capacity of the polymer [52].

Rule of Mixtures, as described in Equation (5), predicts that the combination of a polymer and fiber should create an increased specific heat capacity if the specific heat capacity of the fiber is greater than the specific heat capacity of the polymer itself. In the case of the materials in question, the $C_{p}$ of the matrix is around 0.85 J/g-°C for ABS, 1.12 J/g-°C for PC, and 1.07 J/g-°C for PETG, while the $C_{p}$ values of the carbon and glass fibers are 0.93–0.95 J/g-°C and 0.78–0.94 J/g-°C [56], respectively. Another difference in thermal properties may explain the increase in $C_{p}$. Carbon fiber (CF) is thermally conductive (approx. 10 W/m°C) while glass fiber (GF) is an insulator (<1 W/m°C). The GF, as an insulator, appears to retain heat energy within the material, while CF, as a conductor, dissipates this energy. Since the conductive fibers dissipate the heat, increasing the temperature of the material will require an increased amount of energy. Although more testing should be done, the results show that composites using insulation fibers either decrease $C_{p}$ from the neat polymer state or increase $C_{p}$ at a lower rate compared to conductive materials due to their lower $C_{p}$ values, as seen by others [11,42,57].

The results of Table 4 show that neat materials experience a larger $\Delta C_{p}$ at $T_{g}$ compared to the fiber-filled counterpart. The difference can be seen in a decrease of 21.9% for ABS, 22.7% for PC, and 30.0% for PETG. This phenomenon was also demonstrated by Romanova et al. [41], where the neat ABS and PC have larger $\Delta C_{p}$ values and show a decrease once blended at different ratios with each other. The decrease in $\Delta C_{p}$ at $T_{g}$ signifies the requirement for less energy needed to create the endothermic event.

### 3.3. TMA Results

#### 3.3.1. Strain vs. Temperature

**Figure 8:**
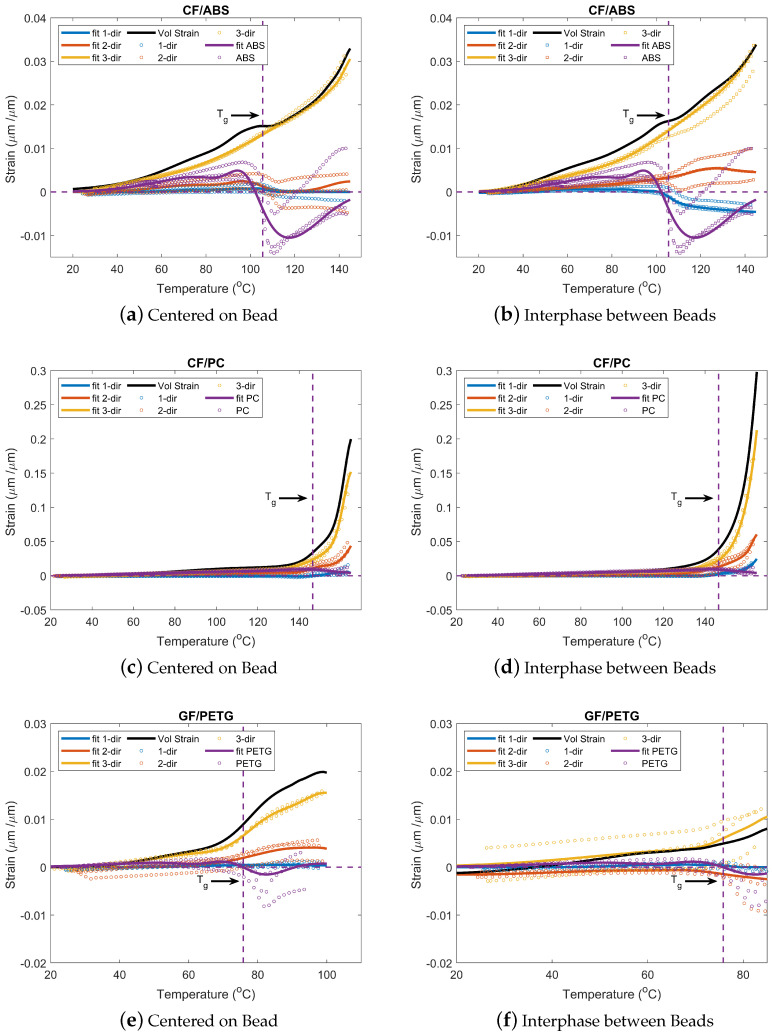
Strain as a function of temperature for (**a**) CF/ABS COB; (**b**) CF/ABS IBB; (**c**) CF/PC COB; (**d**) CF/PC IBB; (**e**) GF/PETG COB; and (**f**) GF/PETG IBB.

Figure 8 shows the strain data as a function of temperature for all the materials tested. This includes both neat and fiber-filled materials. Moreover, strain data for two locations in the printed parts are also shown for the fiber-filled materials. In this case, since all the materials are amorphous, the glass transition temperature can be determined. TMA data provide a quantitative measure of the material deformation given a temperature input. Using this technique, thermal events can be captured.

As shown in Figure 8, the glass transition temperature determined from the DSC test is indicated for reference purposes. For all the locations and materials tested, a change in strain is observed as temperature increases. The difference in strain is related to the change in volume arising from the material changing from its solid state to its glassy state [58]. As observed, there is a change in slope at the reference $T_{g}$ value, and the TMA captures it. Since the DSC measurement captures a thermal event and the TMA captures a mechanical response, calculated $T_{g}$ values are different [59].

**Figure 9:**
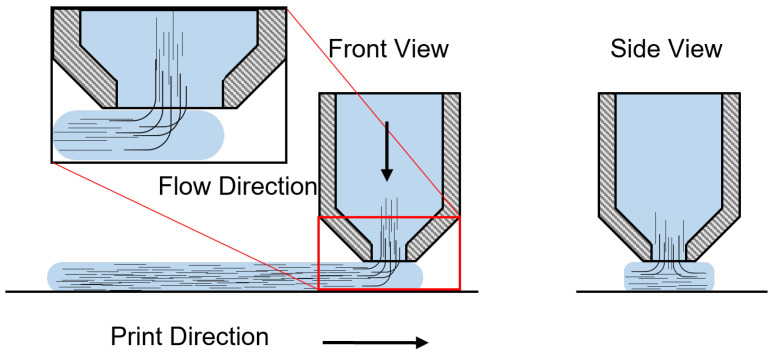
Schematic of fiber alignment during deposition of extruded beads.

Deformation behavior is similar for all the materials. Lower deformation is observed along the bead (1-dir), followed by across the bead (2-dir) and through bead thickness (3-dir), respectively, resulting from the fiber orientation [60]. These results were expected as fibers tend to align in the flow direction, meaning that fibers are primarily oriented in the bead direction. Due to the transversal flow during deposition of the bead, there is partial orientation in the 2-dir. As there is low fiber alignment in the 3-dir, the deformation is higher. Similar results have been found by others [11,61,62,63,64]. A visual representation of this complex phenomenon can be found in Figure 9. Deformation is affected by the fibers in both the 1-dir and 2-dir, while, in the 3-dir, there is a negligible effect of the fibers.

The ABS dimension change results are similar in magnitude to the values found by Billah et al. [11], where TMA results in the transverse and longitudinal direction are compared for CF/ABS, GF/ABS, and neat ABS. Results also show that strain values are affected by the position of the extracted sample. While retaining a general alignment in the bead direction, the local fiber alignment varies across the cross-sections of the printed bead. This indicates that samples centered on the bead will possess lower strain values in the 1-dir than samples at the interphase between beads. Samples at the interphase between beads will have a more complex fiber orientation distribution, affecting the deformation behavior of the material. The fibers constrain the expansion/shrinkage of the sample during the temperature change [65]; this behavior is observed for all three materials tested as shown in Figure 8. Moreover, samples at the interphase between beads will have a different fiber distribution due to the edge flow. They will induce a more complex interaction while deforming, which may lower or increase deformation depending on print parameters [65].

When comparing the fiber-filled materials with the neat materials used in this study, it must be noted that the fiber-filled materials were printed over a year and a half prior to testing. When comparing the neat material with the fiber-filled materials for all the cases, these are within the same magnitude of strain before $T_{g}$. A significant change in strain is observed for temperatures above $T_{g}$. During the TMA test, a contraction behavior for CF/ABS and GF/PETG after reaching its glass transition temperature is observed. This behavior has been found by Cugini et al. [66] and Monnier et al. [67]. The authors attribute this behavior to physical aging, which affects the free volume of the materials. According to Struik [68], physical aging is a gradual continuation of the glass formation that sets around $T_{g}$. Therefore, it can affect the temperature-dependent properties, which change drastically from solid to a glassy state. Physical aging in polymers is associated with a slow loss of free volume that has been trapped in the polymer microstructure after a quenching process (rapid cooling) below $T_{g}$. As the printed material is cooled from the melt state to ambient temperature, it changes state faster than it achieves thermodynamic equilibrium. Once in the solid (glass) state, sufficient mobility exists for it to continue undergoing structural changes towards a state of equilibrium [66]. This state change can have a profound effect on mechanical behavior.

Moreover, the materials themselves often have variations in material properties that can affect the final mechanical properties. This can be caused by the use of additives (i.e., pigments for coloring), different combinations of manufacturers/suppliers/vendors [69], and batch number. The location of the sample within the printed part will also produce a difference in results. The difference in values will result from the temperature gradient within the part, layer time, and cooling rate. These parameters affect the molecules within the part and can produce thermal events as shown in Figure 8.

#### 3.3.2. Volumetric Strain Calculation

A Gaussian function was fitted to the strain data using the MATLAB fitting function to calculate the volumetric strain from the strain data. Equation (6) shows the general form of the Gaussian function. Here, $a_{i}$ has units of μm/μm, and $b_{i}$ and $c_{i}$ have units of °C.

(6) $$\varepsilon \left(T\right)=\sum_{i=1}^{\infty }a_{i}e^{-\left({\left(\frac{\left(T-b_{i}\right)}{c_{i}}\right)}^{2}\right)}$$

Due to the data acquisition frequency, a fitting was performed to capture deformation in the three principal directions at a given temperature for the fiber-filled systems. The fitting parameters for all the directions and locations tested can be found in Table A1, Table A2, Table A3 and Table A4. A similar procedure was done for the neat materials. With the fitted models, the volumetric strain of each fiber-filled system was calculated using Equation (4) over a temperature range and is shown in Figure 8. It is important to mention that the $R^{2}$ value for some of the materials is low. However, the fitted model represents the strain behavior during a temperature change within the tested temperature range. A greater $R^{2}$ value can be achieved by using a higher order of the Gaussian function or by using a custom equation to model the individual behavior. For this work, the selected function order serves the purpose of representing the strain behavior.

The computed volumetric strain physically represents the material response during a temperature change. The volumetric strain for all the materials is within the same magnitude as the strain in the 3-dir. This is lower than an isotropic material where the volumetric strain is three times the linear strain. This, in part, is due to the influence of the fibers within the fiber-filled material. There is high fiber alignment in the 1-dir and significant fiber alignment in the 2-dir. The strain is significantly lower than in the 3-dir. When adding all the strains as shown in Equation (4), the effect of the strain in the 1-dir and 2-dir can be neglected. The impact of the fiber orientation can be observed for both the sample locations (centered on the bead and interphase between beads). As the fiber alignment will be dependent on print parameters, the volumetric strain is expected to change according to these parameters.

#### 3.3.3. CTE vs. Temperature

**Figure 10:**
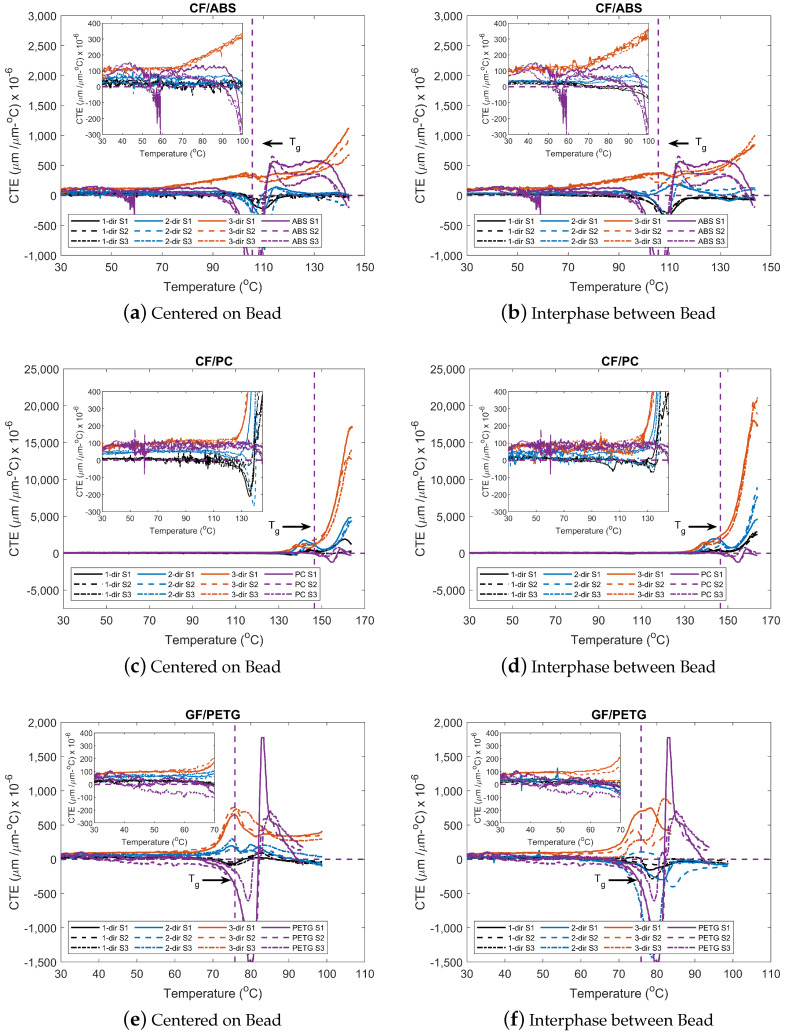
Coefficient of thermal expansion for (**a**) CF/ABS COB; (**b**) CF/ABS IBB; (**c**) CF/PC COB; (**d**) CF/PC IBB; (**e**) GF/PETG COB; and (**f**) GF/PETG IBB. S1, S2, and S3 correspond to sample 1, sample 2, and sample 3 respectively.

The CTE values for each material, sample location, and fiber orientation were computed from the TMA results using the MATLAB gradient function. The gradient was calculated for both the strain and temperature data (shown in Figure 8), with a uniform spacing of 15 points. The data were then smoothed using the smoothdata function with a moving median method and a window of 30. The spacing and window were selected to calculate the CTE for each temperature increment. Each set of TMA tests was performed on three specimens to verify the test’s repeatability. The CTE plots for each material are shown in Figure 10. Selected CTE measurements for the three materials at temperatures before and after the glass transition temperature are presented in Table 6, Table 7 and Table 8.

A trend is observed for all the fiber-filled materials and locations tested. At a temperature of 30 °C (close to ambient temperature), results in the 1-dir possess the lowest CTE values followed by the 2-dir and 3-dir, respectively. The results reinforce the statement of having preferential alignment of fibers in the 1-dir. Moreover, these results support the claim of having orthotropic properties within the printed bead. As shown in Table 6, Table 7 and Table 8, CTE values for the two locations of the fiber-filled systems and the neat material are within the same magnitude, respectively. Moreover, the values obtained for the 3-dir and the neat material at a temperature of 30 °C are in accordance with those found in the literature. These values correspond to the CTE value of the neat material for ABS [70,71], PC [70,71], and PETG [70,72,73] as found in the literature. At temperatures near $T_{g}$, negative CTE values are captured. A negative CTE value corresponds to the contraction of the material at these elevated temperatures. As temperature increases, the free volume of the material also increases, allowing the recovery of the polymeric material [74].

As the glass fibers and carbon fibers have lower CTE values, 4.9–5.1 × 10${}^{-6}$/K for glass fiber [75] and 1.6–10 × 10${}^{-6}$/K for carbon fiber [76], these tend to control the polymer matrix’s deformation (expansion or contraction) during a thermal event. These effects are especially pronounced in the CTE values in the 1- and 2-directions of the printed material, resulting from the flow behavior during deposition. The preferential alignment of fibers during deposition results in significantly lower CTE values in the 1-dir and 2-dir than those in the 3-dir. These effects are generally seen in all three of the materials evaluated in this study and are present both in samples taken from the center of the bead and in samples taken from the interphase between beads.

For some conditions and materials, the CTE values decreased as temperature increased. This event can be attributed in part to the physical aging of the fiber-filled material. As the temperature increases, the enthalpy recovery causes the sample to shrink, gaining mobility as it reaches and surpasses the $T_{g}$ of the respective material [74]. In our results, this behavior is most pronounced for the CF/ABS and GF/PETG. The thermal instability arises from the thermal history of the sample during print. During the cooling of the sample, the material goes from the extruding temperature to ambient temperature in an uncontrolled way. This prevents the material from being in thermodynamic equilibrium. During the TMA heating, the free volume increases, allowing increased mobility of the molecules. The change in CTE is more significant as it reaches $T_{g}$ (solid to glassy state). The enthalpy subsequently recovers to reach the thermal equilibrium, and the sample shrinks. However, the event increases in complexity due to the effect of the fibers, which constrains the movement of the molecules. The decrease in CTE is observed during the $T_{g}$ transition. After $T_{g}$, the CTE value increases with temperature for all the materials, corresponding to the increase in volume during heating.

The CTE results show the benefit of utilizing fillers to control deformation during the heating and cooling process. As the fillers control the material’s expansion, these reduce the thermal stresses and subsequently warpage of the printed part. The results obtained are valid for the conditions presented in Table 2, and the processing parameters presented in Section 2.1.1. It is expected that the thermo-mechanical response of a fiber-filled printed part will be dependent on the processing parameters, bead geometry, and length of filler utilized. The results provide a better understanding of the fiber effects on the CTE response. The authors hope that the results found here serve as a guideline for designers and modelers in general.

#### 3.3.4. Implementation of CTE Data for Numerical Modeling

This section illustrates a procedure for implementing the CTE data for simulation tools. A material and location within the printed part will be used to represent the process. It is often difficult to utilize and implement raw data in numerical modeling. This can produce convergence problems, and it can be computationally expensive. For this reason, a Gaussian function of 4th order, shown in Equation (7), was fitted to the experimental data in Figure 10c.

(7) $$CTE\left(T\right)=\sum_{i=1}^{\infty }d_{i}e^{-\left({\left(\frac{\left(T-f_{i}\right)}{g_{i}}\right)}^{2}\right)}$$

**Figure 11:**
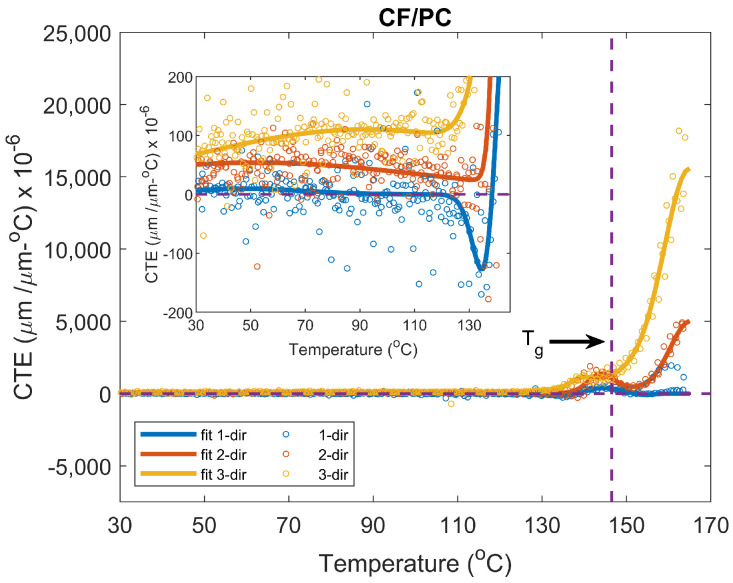
CTE comparison of experimental data and fitting function.

Here, $d_{i}$ has units of (μm/μm-°C ) × 10${}^{-6}$ and $f_{i}$ and $g_{i}$ have units of °C. The function represents a model that can predict the thermal behavior; in this case, the CTE as a function of temperature, in an accurate way. Figure 11 shows the experimental data and the fitting function for CF/PC centered on the bead. Table 9 shows the fitting parameters of the function corresponding to Figure 11.

The function used for the data shows a good fit with $R^{2}$ values greater than 0.93. The excellent fit can also be seen in Figure 11. For ease of visualization, the Matlab ‘MarkerIndices’ option was enabled to plot every 35 data points equal to approximately a degree difference between each data point. The model can capture the variation of CTE values over a temperature range. It is good to mention that the model is valid for the given temperature range (30–165 °C). Moreover, it is recommended to select a function that best represents the behavior of the experimental data as a Gaussian function may not be suitable for all data sets.

The implementation presented in this section aims to help modelers and facilitate the accurate prediction of thermo-mechanical properties during a given manufacturing process. Given the obtained model, the CTE can be calculated over a range of temperature intervals. At the same time, the CTE can be calculated for all three directions at the same temperature value. This implementation facilitates the interpolation process as the model can be implemented through analytical input or by supplying the data in a table form.

## 4. Conclusions

The thermo-mechanical properties of CF/ABS, CF/PC, GF/PETG, and their neat counterparts were successfully characterized for large format polymer extrusion-based additive manufacturing. A roadmap was presented, including equipment and techniques that determine material behavior resulting from large format polymer extrusion-based additive manufacturing. It can be concluded that:

1. TGA results showed an increase in the remaining percent mass at $T_{crit}$ and $T_{term}$ for fiber-filled polymers versus neat polymers. For ABS and PETG, the increase in percent mass is similar to the fiber mass percentage. The PC had a 10% increase with the addition of CF compared to the neat counterpart. The difference is believed to result from additives placed by the manufacturer in the PC materials or char. $T_{deg-onset}$ of ABS varied minimally with the addition of CF, while the addition of fibers to the PETG and PC led to a decrease in $T_{deg-onset}$.
2. DSC results showed fibers’ influence on the composite $T_{g}$ values. The specific heat capacities increased along with temperature and changed with the addition of fibers and increased with conductive carbon fibers, while they decreased when insulator fibers such as glass are used. The variances in the neat materials seem to have little effect on the outcome of composite fiber-filled materials.
3. The strain data show that preferential fiber alignment is present in the parts. Samples showed lower deformation in the 1-dir followed by the 2-dir. The 3-dir possessed the highest deformation. This trend was observed for temperatures below and above $T_{g}$. Near $T_{g}$, an event where the materials possess a negative strain occurs. This is attributed to the free-volume and phase change process going from solid to glassy.
4. CTE results showed that neat materials are similar in magnitude to that of the 3-dir, corresponding to low fiber alignment in the 3-dir. CTE values of samples taken from the interphase between beads are greater than those centered on the bead and are partly due to the complex local fiber alignment at the interphase between the beads. Results show orthotropic behavior in the 3D printed materials. CTE results were consistent with values found in the literature. A Gaussian function was best suited to describe the CTE behavior of the materials used but may not be adequate for all data sets.

Designers and modelers need to understand the effect of fiber orientation and its impact on mechanical properties. Accurate prediction of material properties is necessary when modeling and comparing simulation and experimental results.

## Figures and Tables

**Table 1:** Material technical information.

Type of Material	Supplier	Family	Code	Fiber Content wt. %	Common Name
Neat	JABIL	-	ABS 1400	-	ABS
	SABIC	SABIC^®^	PC Resin PC0703R	-	PC
	TECHMER	HIFILL^®^	PETG 17043DP	-	PETG
Fiber Filled	TECHMER	ELECTRAFIL^®^	ABS 1501 3DP	20	CF/ABS
	TECHMER	ELECTRAFIL^®^	PC 1705 3DP	25	CF/PC
	TECHMER	HIFILL^®^	PETG 1701 3DP	30	GF/PETG

**Table 2:** Material processing parameters.

Material	Heating Zones (°C)				Feed Rate (mm/min)	Screw Speed (RPM)	Layer Time (s)
	1	2	3	4			
CF/ABS	200	220	240	250	4870	99	80
CF/PC	270	305	305	315	4875	68	80
GF/PETG	220	240	270	275	1300	25	260

**Table 3:** Summary of TGA results. $T_{crit}$ refers to the temperature at the end of the single-stage degradation, while $T_{term}$ is the temperature at the end of the test. The degradation temperature was measured for all the samples as the on-set temperature of the mass loss percentage versus temperature curve ($T_{deg-onset}$) and peak of the derivative of the mass loss percentage versus temperature curve ($T_{deg-peakDTG}$).

Material	Percent Mass at $T_{\mathrm{crit}}$	Percent Mass at $T_{\mathrm{term}}$	$T_{\deg-\mathrm{onset}}$ (°C)	$T_{\deg-\mathrm{peak}\mathrm{DTG}}$ (°C)
ABS	2.82%	0.79%	384.26 ± 1.43	407.79 ± 1.34
CF/ABS	23.27%	22.46%	384.15 ± 1.69	410.77 ± 1.84
PC	29.02%	25.64%	480.29 ± 9.09	499.29 ± 5.53
CF/PC	40.49%	36.25%	450.91 ± 3.86	481.97 ± 1.07
PETG	8.57%	5.85%	387.61 ± 1.68	413.67 ± 3.54
GF/PETG	37.46%	35.57%	375.10 ± 3.19	396.24 ± 6.39

**Table 4:** Summary of $T_{g}$ and $C_{p}$ results. The $T_{g}$ was measured as the mid-point temperature ($T_{g-midpoint}$) of the normalized heat capacity versus temperature curve. The change in $C_{p}$ at the glass transition region was also computed. $C_{p}$ values at a temperature of 35 °C and at the test maximum temperature are displayed to show the temperature-dependence of $C_{p}$.

Material	$T_{g-\mathrm{mid}\mathrm{point}}$ (°C)	$\Delta C_{p}$ @ $T_{g}$ (J/g-C)	$C_{p}$ @ 35 °C (J/g-C)	$C_{p}$ @ Maximum Temperature (Table 5) (J/g-C)
ABS	104.32 ± 1.90	0.32 ± 0.03	0.85 ± 0.20	1.71 ± 0.22
CF/ABS	106.57 ± 0.23	0.25 ± 0.02	1.17 ± 0.03	1.97 ± 0.02
PC	149.00 ± 0.19	0.22 ± 0.01	1.12 ± 0.10	1.97 ± 0.12
CF/PC	145.35 ± 0.28	0.17 ± 0.01	1.18 ± 0.27	2.02 ± 0.36
PETG	77.51 ± 0.14	0.26 ± 0.01	1.07 ± 0.03	1.77 ± 0.05
GF/PETG	75.99 ± 0.42	0.20 ± 0.01	0.75 ± 0.01	1.28 ± 0.09

**Table 5:** Maximum temperature for the DSC test.

Material	ABS	PC	PETG	CF/ABS	CF/PC	GF/PETG
Temperature (°C)	250	250	200	250	250	200

**Table 6:** CTE values for CF/ABS and ABS ($T_{g}$ = 104–108 °C).

Location	Direction	CTE (μm/μm-°C × 10${}^{-6}$)			
		30 °C	90 °C	115 °C	145 °C
COB *	1	25.0	13.8	−36.5	−24.2
	2	39.2	42.9	56.3	−56.1
	3	79.9	255	288	903
IBB *	1	18.7	−10.2	−78.2	−34.9
	2	32.4	57.9	199	55.7
	3	84.4	254	328	897
Neat	-	82.0	37.3	500	−165

* Note: COB = Centered on Bead & IBB = Interphase between Beads.

**Table 7:** CTE values for CF/PC and PC ($T_{g}$ = 147–150 °C).

Location	Direction	CTE (μm/μm-°C × 10${}^{-6}$)			
		30 °C	130 °C	150 °C	165 °C
COB *	1	7.81	−48.5	129	540
	2	40.2	22.9	492	4680
	3	71.6	183	2080	14,500
IBB *	1	8.18	−14.8	276	2860
	2	29.0	41.2	775	7040
	3	66.2	210	2850	19,100
Neat	-	62.2	85.8	−246	−121

* Note: COB = Centered on Bead & IBB = Interphase between Beads.

**Table 8:** CTE values for GF/PETG and PETG ($T_{g}$ = 77–78 °C).

Location	Direction	CTE (μm/μm-°C × 10${}^{-6}$)			
		30 °C	70 °C	85 °C	100 °C
COB *	1	20.3	6.07	36.5	−54.5
	2	25.2	85.5	133	−41.3
	3	73.6	188	378	350
IBB *	1	19.8	−5.68	−28.4	−49.6
	2	27.1	−36.4	−148	−83.0
	3	63.0	135	530	-
Neat	-	65.1	−74.7	620	60.7

* Note: COB = Centered on Bead & IBB = Interphase between Beads.

**Table 9:** Gaussian fitting for CF/PC centered on the bead.

Fitting Parameters	CTE (μm/μm-°C) × 10${}^{-6}$		
	1-dir	2-dir	3-dir
$d_{1}$	9.59	53.93	1.42 × 10${}^{4}$
$f_{1}$	48.29	51.73	165.23
$g_{1}$	30.85	88.87	8.53
$d_{2}$	−109.92	5.02 × 10${}^{3}$	1.73 × 10${}^{3}$
$f_{2}$	154.44	165.29	157.24
$g_{2}$	2.65	7.03	15.94
$d_{3}$	−293.24	1.22 × 10${}^{3}$	550.38
$f_{3}$	139.63	143.88	139.14
$g_{3}$	7.91	4.40	3.71
$d_{4}$	609.28	290.40	110.24
$f_{4}$	143.65	151.05	92.15
$g_{4}$	6.10	7.13	89.42
$R^{2}$	0.9693	0.9345	0.9996

## Data Availability

Not applicable.

## Appendix A. Strain vs. Temperature Gaussian Fitting Parameters

**Table A1:** Gaussian fitting parameters for CF/ABS material.

Material	CF/ABS COB			CF/ABS IBB		
Direction	1	2	3	1	2	3
$a_{1}$	4.83 × 10${}^{-4}$	1.70 × 10${}^{-3}$	6.50 × 10${}^{-3}$	1.30 × 10${}^{-3}$	4.80 × 10${}^{-3}$	−8.28 × 10${}^{-1}$
$b_{1}$	104.38	97.83	146.84	101.88	134.31	176.10
$c_{1}$	4.18	9.64	6.30	6.41	46.65	50.63
$a_{2}$	5.78 × 10${}^{-4}$	2.40 × 10${}^{-3}$	8.03 × 10${}^{-4}$	1.30 × 10${}^{-3}$	8.59 × 10${}^{-4}$	9.25 × 10${}^{-1}$
$b_{2}$	96.52	145.11	136.57	91.86	124.24	180.06
$c_{2}$	8.44	13.73	4.64	12.80	9.98	53.49
$a_{3}$	5.35 × 10${}^{-4}$	1.60 × 10${}^{-3}$	2.87 × 10${}^{-2}$	−9.40 × 10${}^{-3}$	7.25 × 10${}^{-4}$	2.10 × 10${}^{-3}$
$b_{3}$	78.20	75.88	176.67	85.76	67.26	61.63
$c_{3}$	17.60	25.16	80.58	5.25 × 10${}^{-4}$	25.24	24.76
$a_{4}$	-	-	-	−2.80 × 10${}^{-3}$	-	-
$b_{4}$	-	-	-	92.95	-	-
$c_{4}$	-	-	-	52.42	-	-
$a_{5}$	-	-	-	2.55 × 10${}^{-5}$	-	-
$b_{5}$	-	-	-	79.97	-	-
$c_{5}$	-	-	-	7.36 × 10${}^{-1}$	-	-
$a_{6}$	-	-	-	3.00 × 10${}^{-3}$	-	-
$b_{6}$	-	-	-	73.87	-	-
$c_{6}$	-	-	-	33.06	-	-
$a_{7}$	-	-	-	−4.20 × 10${}^{-3}$	-	-
$b_{7}$	-	-	-	166.13	-	-
$c_{7}$	-	-	-	51.10	-	-
$R^{2}$	0.3059	0.3836	0.9980	0.9420	0.9835	0.9978

**Table A2:** Gaussian fitting parameters for CF/PC material.

Material	CF/PC COB			CF/PC IBB		
Direction	1	2	3	1	2	3
$a_{1}$	−8.08 × 10${}^{8}$	2.53 × 10${}^{9}$	9.02 × 10${}^{-2}$	9.64 × 10${}^{-2}$	1.74 × 10${}^{-2}$	1.17 × 10${}^{5}$
$b_{1}$	676.11	514.14	165.59	178.93	165.31	345.52
$c_{1}$	102.60	70.05	5.72	11.59	3.61	49.53
$a_{2}$	4.30 × 10${}^{-3}$	5.50 × 10${}^{-3}$	7.68 × 10${}^{-2}$	4.40 × 10${}^{-3}$	0	1.87 × 10${}^{-2}$
$b_{2}$	165.19	149.83	178.17	157.98	151.87	215.52
$c_{2}$	3.10	6.55	26.30	7.64	2.00 × 10${}^{-3}$	100.15
$a_{3}$	1.36 × 10${}^{-2}$	4.30 × 10${}^{-3}$	7.50 × 10${}^{-3}$	2.60 × 10${}^{-3}$	9.71	0
$b_{3}$	163.16	124.46	118.64	149.22	340.55	1.55 × 10${}^{3}$
$c_{3}$	11.67	57.10	48.29	5.89	75.44	269.08
$R^{2}$	0.8752	0.9834	0.9969	0.9870	0.9046	0.9974

**Table A3:** Gaussian fitting parameters for GF/PETG material.

Material	GF/PETG COB			GF/PETG IBB		
Direction	1	2	3	1	2	3
$a_{1}$	1.73 × 10${}^{-4}$	3.27 × 10${}^{-4}$	2.7 × 10${}^{-3}$	0	34.34	5.50 × 10${}^{-3}$
$b_{1}$	91.77	99.36	61.75	80.26	35.48	87.13
$c_{1}$	1.25	3.29	18.29	1.72 × 10${}^{-4}$	12.58	4.70
$a_{2}$	−2.84 × 10${}^{-4}$	−3.96 × 10${}^{-5}$	−3.58 × 10${}^{-2}$	4.64 × 10${}^{-4}$	18.78	3.90 × 10${}^{-3}$
$b_{2}$	98.74	90.81	93.05	69.84	51.62	80.91
$c_{2}$	13.03	2.49	11.78	7.00	6.38	7.08
$a_{3}$	6.53 × 10${}^{-4}$	4.10 × 10${}^{-3}$	4.97 × 10${}^{-2}$	−5.5 × 10${}^{-3}$	18.78	3.70 × 10${}^{-3}$
$b_{3}$	97.17	92.68	93.82	78.98	51.62	72.92
$c_{3}$	35.26	19.58	13.92	66.11	6.38	34.27
$a_{4}$	-	-	-	8.49 × 10${}^{6}$	-	-
$b_{4}$	-	-	-	79.28	-	-
$c_{4}$	-	-	-	−8.9 × 10${}^{-3}$	-	-
$a_{5}$	-	-	-	4.3 × 10${}^{3}$	-	-
$b_{5}$	-	-	-	63.14	-	-
$c_{5}$	-	-	-	34.60	-	-
$R^{2}$	0.2838	0.5530	0.9945	0.0658	0.9067	0.2654

**Table A4:** Gaussian fitting parameters for neat materials.

Material	ABS	PC	PETG
$a_{1}$	−1.35 × 10${}^{-2}$	0	−2.33 × 10${}^{-2}$
$b_{1}$	114.57	273.76	74.02
$c_{1}$	22.77	20.28	11.16
$a_{2}$	7.30 × 10${}^{-3}$	4.40 × 10${}^{-3}$	2.23 × 10${}^{-2}$
$b_{2}$	97.79	143.91	73.37
$c_{2}$	9.45	17.71	10.25
$a_{3}$	4.80 × 10${}^{-3}$	6.40 × 10${}^{-3}$	1.60 × 10${}^{-3}$
$b_{3}$	89.63	115.14	71.99
$c_{3}$	36.50	57.31	34.71
$R^{2}$	0.9424	0.9275	0.9414

## Appendix B. Determination of *T_crit_* and *T_deg_*

**Table A5:** TGA $T_{crit}$ from Data.

Sample Number	Parameter	ABS	CF/ABS	PC	CF/PC	PETG	GF/PETG
1	Temperature (°C)	457.15	464.76	530.17	518.42	449.87	487.27
	Percentage (%)	2.623	22.64	31.10	40.38	8.79	37.42
2	Temperature (°C)	454.49	455.16	557.75	504.43	454.05	481.94
	Percentage (%)	2.867	23.27	28.95	41.4	8.186	38.34
3	Temperature (°C)	455.38	462.13	537.73	526.28	450.48	446.81
	Percentage (%)	2.957	23.90	27.02	39.70	8.734	36.62

**Table A6:** TGA $T_{deg}$ from data.

Sample Number	Parameter	ABS	CF/ABS	PC	CF/PC	PETG	GF/PETG
1	Temperature (°C)	695.57	595.36	595.31	695.33	595.33	595.40
	Percentage (%)	1.359	22.32	27.46	35.95	6.722	36.67
2	Temperature (°C)	695.55	592.98	695.44	595.41	695.37	695.35
	Percentage (%)	0.6329	22.52	26.23	36.90	5.279	37.00
3	Temperature (°C)	695.54	592.98	695.35	695.63	695.37	595.45
	Percentage (%)	0.3774	22.53	23.25	35.89	5.549	33.05
